# Self-emulsifying drug delivery systems: A versatile approach to enhance the oral delivery of BCS class III drug via hydrophobic ion pairing

**DOI:** 10.1371/journal.pone.0286668

**Published:** 2023-06-09

**Authors:** Muhammad Asad, Akhtar Rasul, Ghulam Abbas, Muhammad Ajmal Shah, Imran Nazir

**Affiliations:** 1 Department of Pharmaceutics, Faculty of Pharmaceutical Sciences, Government College University Faisalabad, Faisalabad, Pakistan; 2 Department of Pharmacy, Hazara University, Mansehra, Pakistan; 3 Department of Pharmacy, COMSATS University Islamabad, Lahore campus, Lahore, Pakistan; Central University of Rajasthan, INDIA

## Abstract

Biopharmaceutical classification systems (BCS) class III drugs belongs to a group of drugs with high solubility in gastrointestinal (GI) fluids and low membrane permeability result in significantly low bioavailability. Self-emulsifying drug delivery systems (SEDDS) considered a suitable candidate to enhance the bioavailability of poorly soluble drugs by improving their membrane permeability, however, incorporating hydrophilic drugs in to these carriers remained a great challenge. The aim of this study was to develop hydrophobic ion pairs (HIPs) of a model BCS class-III drug tobramycin (TOB) in order to incorporate into SEDDS and improve its bioavailability. HIPs of TOB were formulated using anionic surfactants sodium docusate (DOC) and sodium dodecanoate (DOD). The efficiency of HIPs was estimated by measuring the concentration of formed complexes in water, zeta potential determination and log P value evaluation. Solubility studies of HIPs of TOB with DOC were accomplished to screen the suitable excipients for SEDDS development. Consequently, HIPs of TOB with DOC were loaded into SEDDS and assessed the log D_SEDDS/release medium_ and dissociation of these complexes at different intestinal pH over time. Moreover, cytotoxic potential of HIPs of TOB and HIPs loaded SEDDS formulations was evaluated. HIPs of TOB with DOC exhibited the maximum precipitation efficiency at a stoichiometric ratio of 1:5. Log P of HIPs of TOB improved up to 1500-fold compared to free TOB. Zeta potential of TOB was shifted from positive to negative during hydrophobic ion pairing (HIP). HIPs of TOB with DOC was loaded at a concentration of 1% (w/v) into SEDDS formulations. Log D_SEDDS/release medium_ of loaded complexes in to oily droplets was above 2 and dissociated up to 20% at various pH within 4 h. Finding of this study suggested that improvement of the lipophilic character of BCS class-III drugs followed by incorporation into oily droplets can be deliberated as a promising tool to enhance the permeation across biological membranes.

## 1. Introduction

Pursuant to biopharmaceutical classification system (BCS), the rate and extent of drug absorption mainly depend on their solubility and permeability through the biological membranes [1]. During recent decades, BCS class-III drugs in particular therapeutic peptides and proteins are focus of interest due to their high therapeutic potential [2, 3]. BCS class-III drugs are hydrophilic compounds and highly soluble in gastrointestinal (GI) fluids but exhibit low permeability across the epithelial cells [4]. Hence, enormous BCS class-III drugs still has to be administered through a parenteral route and often requires the assistance of trained specialists. Therefore, the pharmaceutical industry is mainly focused on the development of oral delivery systems for BCS class III drugs to improve the patient compliance.

Oral bioavailability of BCS class III drugs improved using various alternative approaches such as enteric drug delivery systems, microparticles [5], liposomes [6, 7], and emulsions containing hydrophobic ion pairs (HIPs) [8] have been evaluated. Various factors for instance low drug loadings into the carrier systems, deprived encapsulation efficiencies and deficit of proper scalability inhibit these systems to reaching into the market. Therefore, hydrophobic ion pairing (HIP) emerges as a successful tool for delivering these drugs. HIPs are based on the formation of a neutral complex between a hydrophilic ionized drug and an oppositely charged surfactant which potentially can lead to improve drug lipophilicity resulting in enhanced permeation across biological membranes [9]. Ion pair complexes protect the drug from enzymatic degradation, improve circulation time, controlled release of the drugs, reduce toxicity followed by enhance bioavailability are the robust parameters to develop highly scalable loaded carrier systems having high encapsulation efficiencies. HIP increases the lipophilicity of hydrophilic drugs without altering the drug’s molecular structure [10]. In contrast, the drug solubility in aqueous media such as the GI fluid could be noticeably reduced due to increase in the lipophilicity. However, HIPs failed to progress the oral bioavailability of BCS class III drugs in vivo due to their instability in gastrointestinal (GI) fluid. Due to this instability, HIPs cannot reach to the epithelial membranes in intact form. In order to resolve this issue, SEDDS can be beneficial to administer these HIPs of hydrophilic drugs in intact form to the epithelial membrane resulting in improve their bioavailability. SEDDS are the isotropic mixtures of oils, surfactants, co-surfactants and/or co-solvents forming homogenized oil in water (O/W) emulsion upon dilution with GI fluids [11, 12]. SEDDS exhibits the potential to enhance the oral bioavailability of the drugs as they exhibit the protective effect towards the enzymatic degradation and sulfhydryl barrier on the one hand and improve the permeation across the mucosal barrier of the intestine on the other hand [13, 14]. Moreover, SEDDS seem to be more efficacious delivery systems from industrial point of view due to their ease in production and scale up [15]. Li et al. described that SEDDS can only be efficacious when the loaded HIPs remain in the oily droplets till reached at the site of absorption [16] The release pattern of the loaded HIPs from SEDDS plays a critical role for the effectiveness of these delivery systems. The distribution coefficient (log D_SEDDS/release medium_) can be considered as key factor for drug release as equilibrium is immediately reached between SEDDS pre-concentrate and intestinal fluids [17]. Tobramycin *(TOB)* is a BCS class III drug which is used against gram negative bacterial infections such as peritonitis, pneumonia and urinary tract infections [18]. It is highly polar cationic compound that have poor oral bioavailability thus administer via topical and parental route. Due to its low bioavailability, TOB was chosen as a model drug.

The present study aimed to develop HIPs of TOB in order to deliver through oral route. Various anionic surfactants such as docusate sodium (DOC) and sodium dodecanoate (DOD) were chosen as counter ions. These lipophilic complexes were loaded into SEDDS. The release of the HIPs of TOB form the oily droplets and dissociation of these complexes were estimated at various GI conditions. Furthermore, toxicity study of these HIPs and HIPs loaded SEDDS is also subject of this investigation.

## 2. Materials and methods

### 2.1. Materials

Tobramycin (TOB) was a gift sample from Welmark Pharmaceuticals (Hattar, Pakistan). Docusate sodium (DOC), sodium dodecanoate (DOD), Propylene glycol and acetonitril were obtained from Sigma-Aldrich (Darmstadt, Germany). Peceol (*glycerol monooleate*, *HLB = 3*), Cremophor RH (*castor oil*, *hydrogenated*, *ethoxylated)* and Maisine 35–1 (*glycerol monolinoleate*, *HLB = 1*) were a gift from Gattefossé (Lyon France).

### 2.2. Methods

#### 2.2.1. HPLC analysis

The concentration of TOB was quantified by HPLC using a previously described method [19, 20]. Samples were analyzed by using a reverse phase C-18100 mm× 4.6 mm, 5 μm column as stationary phase. Acetonitrile 50% (v/v) and water 50% (v/v) used as a mobile phase. The flow rate of 1mL/min was used. Sample of 20 μL was injected and concentration of TOB was evaluated at a wavelength of 230 nm.

#### 2.2.2. Preparation of HIPs

HIPs of cationic hydrophilic model BCS class-III drug TOB was prepared using anionic surfactants as counter ions via HIP as previously described with minor modifications [21]. In brief, 1 mM TOB solution was prepared utilizing 0.1 N HCL exhibiting net positive charge. Thereafter, solutions of each anionic surfactant were prepared applying various molar ratios of TOB to surfactant in order to identify the most suitable compounds for HIP as described in **Table 1**. The surfactant solution was added in a dropwise manner to TOB solution at 25°C whilst shaking at 400 rpm for 2 h using thermomixer (Thermomixer Comfort, Eppendorf, Germany) resulting in a cloudy solution indicating HIPs were formed. HIPs were separated by centrifugation at *10*,*500 g* for 10 min using Mini spin Centrifuge (Eppendorf, Germany). The resulting lipophilic complexes of TOB were washed thrice with water and lyophilized (Christ Gamma 1–16 LSC Freeze dryer). The lyophilized HIPs of TOB were stored at -20°C for further use. The precipitation efficiency was estimated using Eq 1:

$$Precipitation\;efficiency\;(\%)=100-\left(\frac{TOB\;concentration\;after\;HIP}{TOB\;concentration\;before\;HIP}\times 100\right)$$ (1)

**Table 1:** Molar ratios for the preparation of HIPs of TOB using various counterions.

Tested Surfactants	TOB (mM)	Molar ratio (TOB: surfactant)
Docusate sodium (DOC) Sodium dodecanoate (DOD)	1 1 1 1 1	1:1 1:3 1:5 1:7 1:9

#### 2.2.3. Lipophilicity (Log P) determination

Log P of TOB and HIPs of TOB were evaluated in n-octanol (lipophilic phase) and in water (aqueous phase) [22]. In brief, 1 mg of TOB or HIPs of TOB was added in 1 mL of n-octanol/water (1:1) and incubated for 24 h at 37°C with 500 rpm shaking. The resulting samples were then centrifuged at 12,500 rpm using Mini Centrifuge (Thermo Fisher Scientific II, USA) for 10 min. Afterward, aliquots of 100 μL were withdrawn from n-octanol phase as well as water phase Aliquots from both phases were diluted with 300 μL of methanol comprising 0.1% (v/v) Trifluoroacetic acid (TFA). The concentration of TOB in n-octanol and in water phases were measured by HPLC as described above. Log P was estimated using the Eq 2:

$$logP_{n‐octanol/water}=log\frac{Concentration\;of\;TOB\;in\;n-octanol\;phase}{Concentration\;of\;TOB\;in\;aqueous\;phase}$$ (2)

#### 2.2.4. Assessment of zeta potential during HIP

Zeta potential of TOB and TOB loaded lipophilic complexes was evaluated during HIP using a method reported by *Zaichik et al*. with slight modifications [23]. 1 mL of 1 mM TOB solution was prepared in 0.1 N HCl and 1 mL of surfactants solution in concentration equivalent to molar ratios of TOB to surfactant were dissolved as described above. The surfactant solution was added to TOB solution and incubated at 25°C with 500 rpm shaking. Zeta potential of TOB and HIPs of TOB at various molar ratios was estimated using Zeta sizer Nano-ZSP (Malvern Instruments, UK).

#### 2.2.5. Solubility studies

Solubility of HIPs in different excipients was determined following a minor modified method described by *Zupančič et al*. [24]. Briefly, 1 mg of lipophilic complexes of TOB was added in various solvents with increasing volumes of each solvent in order to reach dissolution just by vortex mixing. When this was not sufficient, the mixture was stored in thermomixer at 400 rpm at 60°C until complete solubilization was observed. Afterwards, solubility of the HIPs of TOB was visually assessed after centrifugation at 12,500 rpm for 5 min.

#### 2.2.6. Preparation of SEDDS

In order to formulate SEDDS, different amounts of oils, surfactants, co-surfactants and solvents were homogenized, as illustrated in **Table 2**. The excipients of the SEDDS were homogenized by shaking in thermomixer at 500 rpm for 24 h at 37°C in order to form a single phase system. Semisolid components used in the SEDDS formulations were melted before use [20]. Afterwards, SEDDS pre-concentrate (10 μL) were emulsified in 10 mM HEPES buffer (HB) pH 6.8 (990 μL) with shaking at 500 rpm at 37°C for 5 min and 4 h. Samples were then centrifuged at 10,500 rpm for 5 min in order to evaluate visually the stability of emulsions.

**Table 2:** Composition of SEDDS formulations. Values are designated in percentage (% v/v).

*Formulation*	*F1*	*FII*	*FIII*
Cremophor RH	20	20	25
Capryol 90	10	-	-
Labrasol ALF	20	25	15
Miasin 35–1	-	20	-
Peceol	20	-	30
Tween 20	20	25	20
Propylene glycol	10	10	10

#### 2.2.7. SEDDS formulations characterization

Mean droplet size, PDI and zeta-potential of blank as well as 1% w/v TOB-DOC HIPs loaded SEDDS were analyzed by Zeta-sizer Nano-ZSP after emulsification of the pre-concentrate in 10 mM HB pH 6.8 in a dilution 1:100. Each measurement was carried out after incubation of the samples at 500 rpm at 37°C for 5 min and 4 h in thermomixer. For zeta-potential measurements 150 μL of sample were diluted in 850 μL of demineralized water before the measurement.

#### 2.2.8. Dissociation studies

Briefly, 1 mg of TOB-DOC HIP at a molar ratio of 1:1 was dissolved in 500 μL of 10 mM HEPES buffer (HB) comprising 138 mM NaCl, 10 mM glucose, 5 mM KCl 1 mM Mg Cl_2_ and 2 mM CaCl_2_ at pH (6, 6.8 and 7.4). Then, the samples were incubated at predetermined time points (2 and 4 h) at 500 rpm and 37°C. The resulting mixtures were centrifuged for 10 min at 12,500 rpm and the amount of TOB from dissociated complex was estimated in supernatant solution using HPLC as described above.


$$Dissociation\;(\%)=\left(\frac{amount\;of\;TOB\;in\;supernatant}{amount\;of\;TOB\;in\;HIPs}\right)\times 100$$ (3)

#### 2.2.9. Distribution coefficient (log D_SEDDS/release medium_) estimation

The release behavior of the lipophilic complexes of TOB from oily droplets was estimated by evaluating their log D_SEDDS/release medium_ utilizing earlier reported method [17]. Log D_SEDDS/release medium_ was measured by calculating the solubility of lipophilic complexes in SEDDS pre-concentrate on the one hand and in the release medium on the other hand in a separate manner. The amount of HIPs of TOB were dissolved increasingly in 10 μL of SEDDS pre-concentrate, whereas 1 mg of HIPs were added in the release medium at 60°C for 4 h while shaking at 400 rpm. As described above, solubilization of the lipophilic complexes was estimated visually after centrifugation (12,500 rpm) for 5 min. Furthermore, log D_SEDDS/release medium_ was estimated using Eq (4):

LogDSEDDSreleasemedium=log(maximumsolubilityofHIPsofTOBintheSEDDSpre−concentratemaximumsolubilityofHIPsofTOBinthereleasemedium) (4)

#### 2.2.10. Cytotoxic potential determination-resazurin assay

Cytotoxicity of TOB, HIPs of TOB with DOC and SEDDS loaded with HIPs of TOB at a concentration of 1% w/v was evaluated following the resazurin assay [8]. In brief, human colorectal adenocarcinoma-derived cells (Caco-2) were incubated at 37°C under 5% CO_2_ and 95% relative humidity environment for 14 days after seeding in a 24-wells plate with a density of 25,000 cells per well in 500 μL of minimum essential red medium (MEM). The medium was changed first time after 24 h and, then, at alternative days until a complete cells monolayer was obtained. At the time of analysis, the monolayer of the cells were washed twice with preheated 10 mM HB at pH 6.8. Thereafter, preheated test solutions of TOB and TOB-DOC HIPs in 10 mM HB pH 6.8, TOB-DOC HIPs loaded SEDDS (1% w/v) at different dilutions (1:100, 1:500 and 1:1000) in 10 mM HB, positive control (10 mM HB) and negative control (Triton X® 100 in a 0.5% v/v) were added in triplet to the cell culture plate in 500 μL of volume per well. The samples were than incubated for 4 h in abovementioned condition. Afterwards, the supernatant was removed and cells were washed twice with preheated HB before incubation with 2.2 mM resazurin solution at the same conditions for 3 h. Then, fluorescence of the supernatant from each well was measured at 540 nm of excitation wavelength and 590 nm of emission wavelength and cell viability was determined using Eq (5):

$$Cell\;viability\;(\%)=\left(\frac{fluorescence\;intensity\;of\;treated\;cells}{fluorescence\;intensity\;of\;untreated\;cells}\right)\times 100$$ (5)

### 2.3. Statistical data analysis

Statistical data analysis was accomplished using the GraphPad Prism 6 software. The ONE-way ANOVA test and Bonferroni test were used to compare more than 2 mean values and post hoc multiple comparisons test respectively. *p*<0.05 was set as a minimum level of significance. Results were demonstrated as the mean of minimum 3 experiments ± standard deviation (SD).

## 3. Results and discussions

### 3.1. Preparation of HIPs

**Fig 1:**
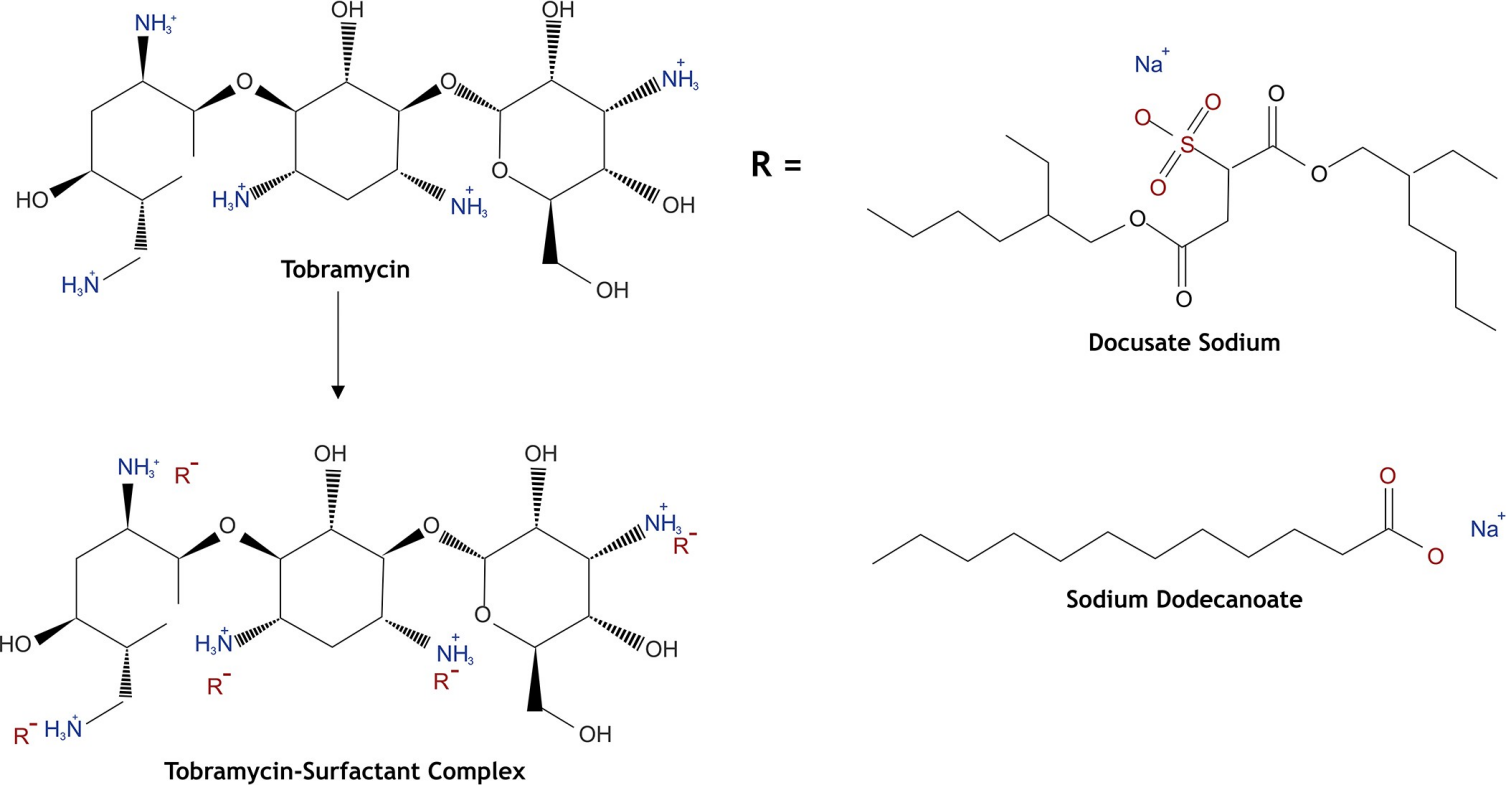
Hydrophobic ion pairing of tobramycin utilizing lipophilic anionic counter-ions.

**Fig 2:**
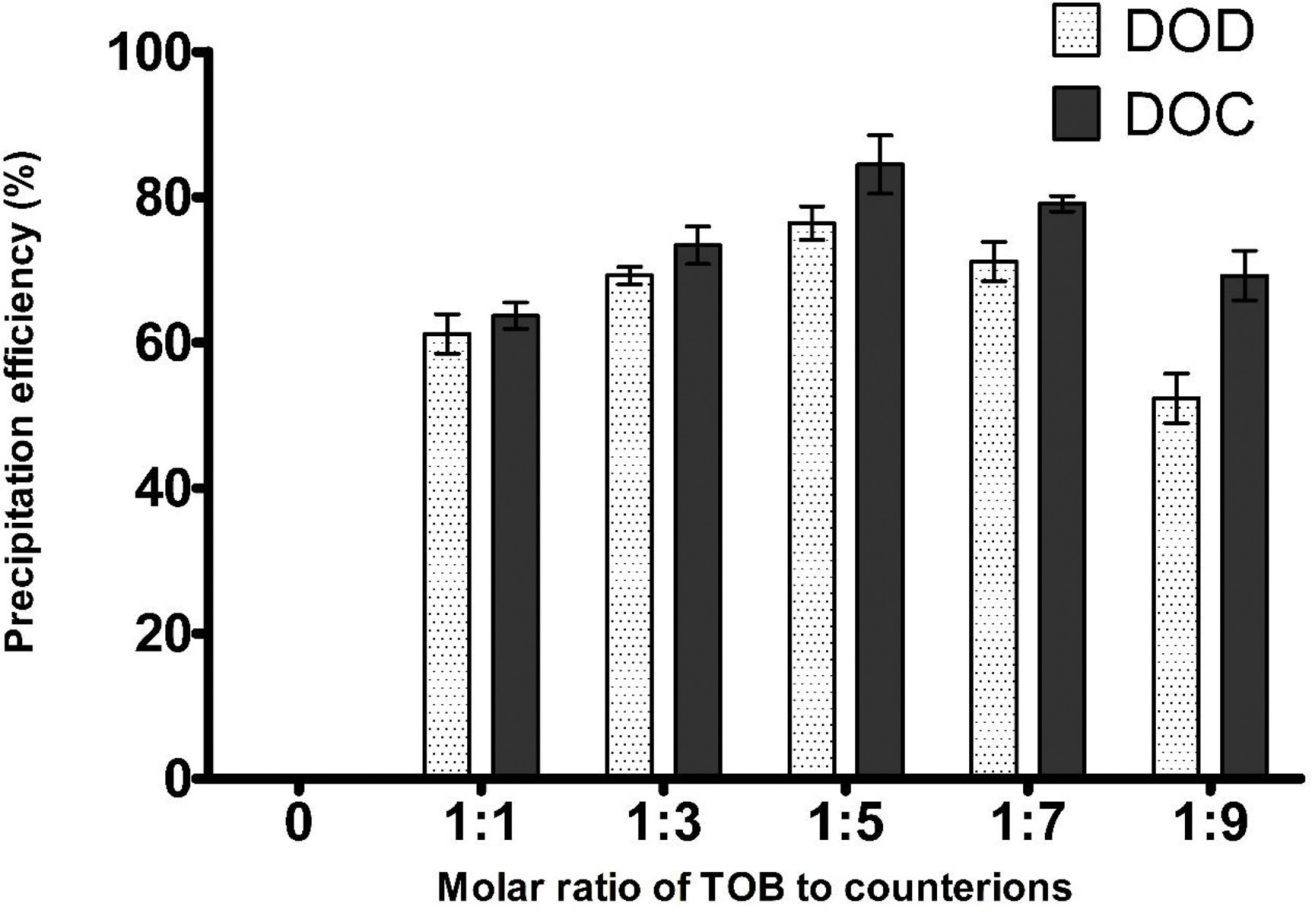
Precipitation efficiency of TOB with surfactants at various molar ratios.

Precipitation efficiency could not reach the 100% probably because intra-molecular hydrogen bonding can occur, therefore, the interaction between drug and surfactant can be reduced. As according to theory, the maximum complexation should occur at equimolar ratio of cationic and anionic charges. Therefore, the highest precipitation efficiency of HIPs of TOB should be registered at molar ratio 1:5, because TOB exhibit five positive charges due to the presence of–NH_3_^+^ and surfactants in solution exhibit one negative charge as illustrated in **Fig 1**. The ion pair formation is primarily based on columbic force which is directly proportional to the magnitudes of the charges [25]. The impact of surfactants exhibits sulfonic and carboxylate moieties were estimated on complex formation as mentioned in Table 3. Although this, as illustrated in **Fig 2**, the results obtained for all surfactants showed that precipitation efficiency at molar ratio 1:5 is much higher than the one at molar ratios 1:7 and 1:9. Previous studies also reported the similar behavior of the complex formation of hydrophilic in particular BCS class III drugs using various counter ions and lipophilicity of the formed complexes did not increased beyond the maximum stoichiometric molar ratio [26–28]. The reason of this behavior can be referred to the fact that HIPs formation can be caused also by other interactions as the hydrophobic ones, which can occur between the ammonium group of TOB and the oxygen atoms in the surfactant structure. Moreover, a decrease in precipitation efficiency above molar ratio 1:5 was might be due to the micelles formation. Nazir et al. reported that the complex formation of leuprolide using non-ionic surfactant sucrose stearate occurred due to the hydrophobic H-bond pairing indicating that H-bonding may also impact on the strength of the ion pairing [29].

**Table 3:** Properties of anionic surfactant used for HIP of TOB.

Surfactant	Chemical formula	Molecular weight	Log P	pKa	Chemical group
Docusate sodium (DOC)	C_20_H_37_NaO_7_S	444.6	4.36	-0.75	sulfonate
Sodium dodecanoate (DOD)	C_12_H_24_NaO_2_	223.3	5.24	4.95	monocarboxylate

### 3.2. Lipophilicity (Log P) determination

**Fig 3:**
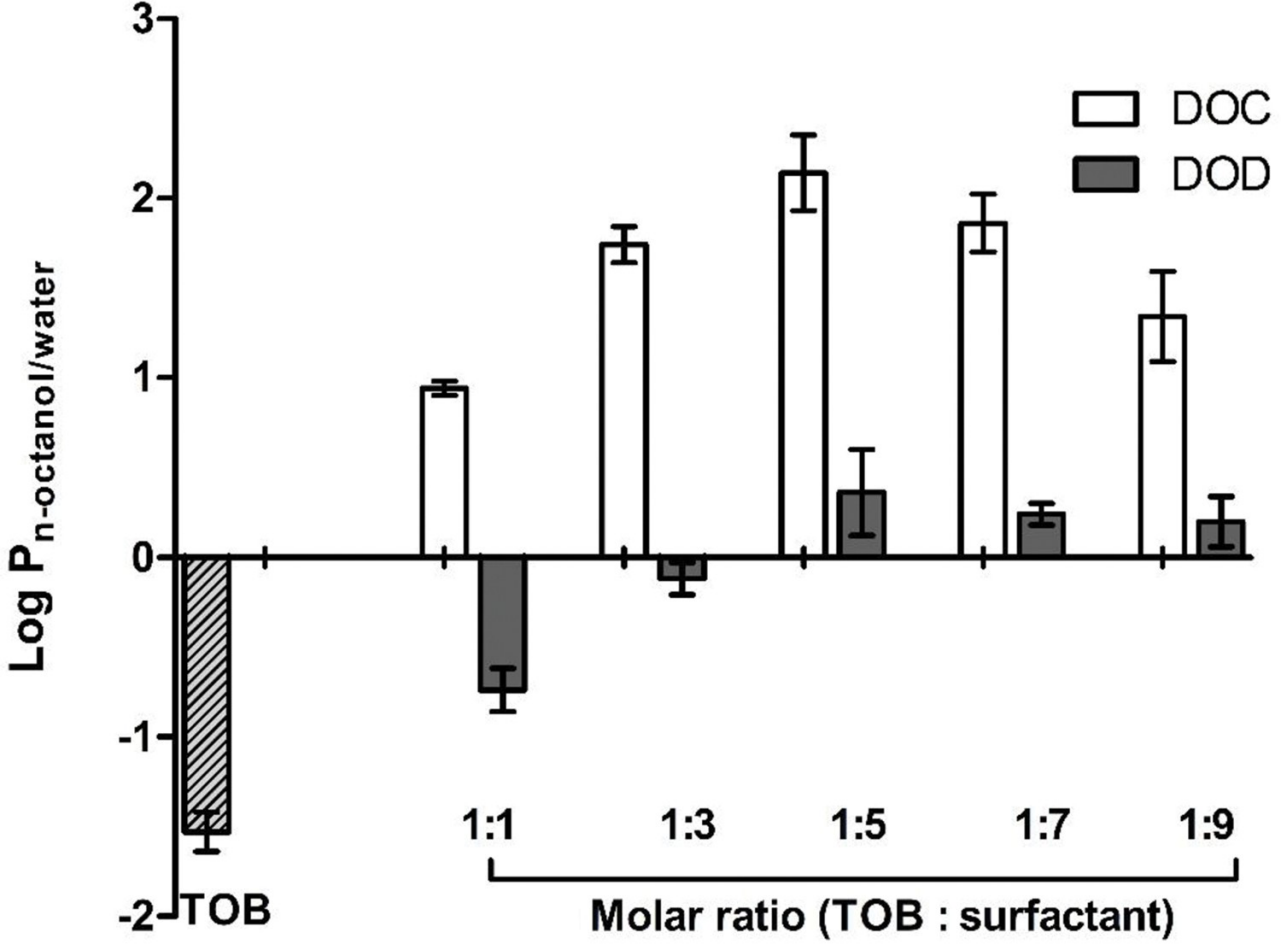
Log P_n-octanol/water_ of TOB and HIPs of TOB.

The diffusion of drugs across the absorption membrane of GI tract is based on the capability of the drugs to cross phospholipidic bilayer of epithelial membranes. According to this, compounds with hydrophobic properties should permeate easier across the biological membranes than molecules with hydrophilic character [30]. Enhancing the lipophilic behavior of hydrophilic ionizable drugs via HIP has efficiently increased their ability to permeate across the epithelial membranes [10]. Miller et al. reported that the permeation of phenformin was improved 4.9-fold across the Caco-2 cell monolayers by enhancing its lipophilic character using 1-hydroxy-2-naphtoic acid as counter ion [31]. Furthermore, a high lipophilicity of HIPs is important to easily incorporate the complex into SEDDS [32]. Log P_n-octanol/water_ values of TOB and HIPs of TOB at various molar ratios are shown in **Fig 3**. Among the tested surfactants, DOC significantly improves the lipophilicity at various molar ratios. The capability of the counter ions to improve the lipophilicity of the complexes is directly related to their ionic strength (pKa). As the DOC tightly bound to the TOB due to its lower pKa (high acidity) compared to DOD resulting in the formation of the more lipophilic complexes. These results are in agreement with a previously reported study [26].

As TOB is a water-soluble compound, its concentration would be higher in aqueous phase than in the organic one, as shown by the negative value of log P_n-octanol/water_. In contrast, the same molecule complexed with different surfactants demonstrated a highly positive log P_n-octanol/water_ in case of all surfactants due to the development of a non-ionic and more hydrophobic complex as shown in **Fig 3**.

### 3.3. Assessment of zeta potential during HIP

**Fig 4:**
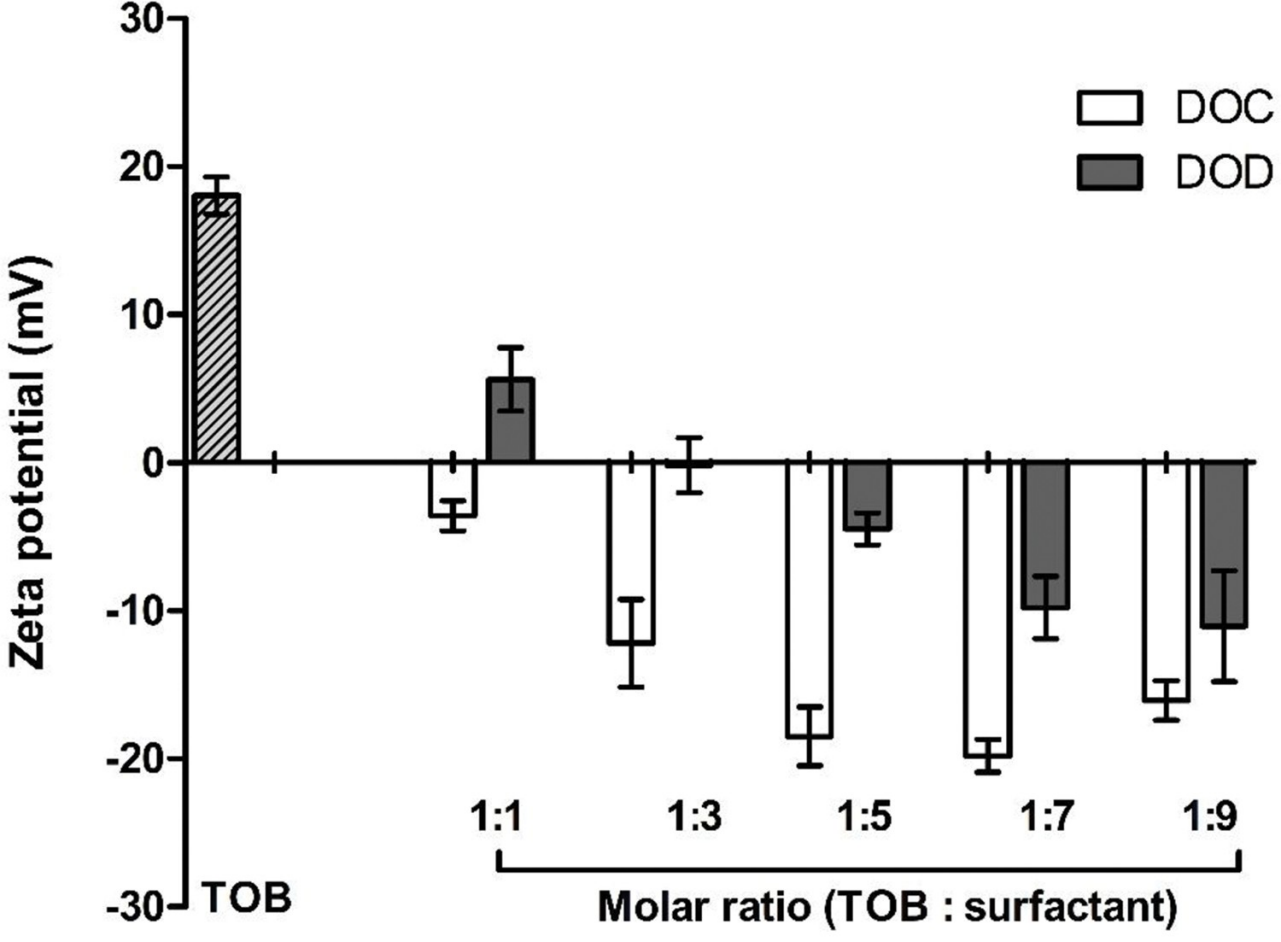
Zeta potential (mV) of HIPs at various molar ratios. Indicated values are mean of three experiments ± SD.

The estimation of zeta potential was required to evaluate the surface charge of TOB or HIPs during the formation of the HIPs. In case of non-complexed TOB, the parameter should be characterized by a positive value due to the presence of–NH_3_^+^, whereas a negative value of zeta potential was registered after HIP because of the arrangement of anionic surfactants on the surface of the drug as depicted in **Fig 4**. Considering precipitation efficiency results, DOC is the surfactant with the major ability to interact with the drug for HIPs formation. As zeta potential is a measure of the drug surface charge, more complexed the drug is with anionic surfactants. Therefore, a decrease in zeta potential value during HIP of TOB with anionic surfactant was observed due to the complexation of–NH_3_^+^ with anionic surfactants. These results of the zeta potential shifting of TOB during HIP are in agreement with a study conducted by Griesser et al. describing a strong correlation of the anionic surfactants on the shift in the zeta potential of the peptides during HIP [21].

### 3.4. Solubility studies

The most efficient log P of TOB was achieved with DOC at a molar ratio of 1:5 and a significant shift in the zeta potential during HIP was also observed with DOC at similar molar ratio. Therefore, HIPs of TOB with DOC at a molar ratio of 1:5 was chosen for further studies. The solubility of HIPs of TOB was evaluated to choose the appropriate oils, surfactants and solvents to develop a stable SEDDS formulation. Excipients with different values of dielectric constant and hydrophilic-lipophilic balance (HLB) were tested. Excipients used and results of solubility studies are depicted in **Table 4**. A visual determination of solubility was performed, because the method is less complicate and time consuming [9]. As the HIPs are more soluble in excipients having high dielectric constant, however, lipophilic complexes of TOB was also solubilized in solvents having low dielectric constant as shown in **Table 4** indicating that lipophilicity of the drugs significantly improved after complexation.

**Table 4:** Solubility studies of HIPs of TOB with DOC at molar ratio of 1:5 in different SEDDS excipients.

Excipient	Chemical name	Functionality	Dielectric constant	HLB value	Solubility of HIPs of TOB with DOC (%)
Cremophor RH	Hydrogenated castor oil	Non-ionic surfactant	--	14	5
Capryol 90	propylene glycol monocaprylate type II	Non-ionic surfactant	14.1	--	6.67
Labrasol ALF	caprylocaproyl macrogol-8 glycerides	Non-ionic surfactant	8.1	12	6.67
Miasin 35–1	glycerol/glyceryl monolinoleate	Oily vehicle	3.4	1	3
Peceol	glycerol mono-oleates type 40	Oily vehicle	3.5	1	3
Tween 20	polyethylene glycol sorbitan monolaurate	Non-ionic surfactant	--	16	6.67
Propylene glycol	propane-1,2-diol	Solvent	32	--	16

### 3.5. SEDDS preparations

Surfactants, co-surfactants, oils and solvents to be used in the formulation of SEDDS have been selected based on their solubility studies. Different concentrations of oils, surfactants and co-solvents were mixed to develop SEDDS formulations. The chosen surfactants used for the SEDDS formulations are safe, efficient and biocompatible in particular Cremphor RH [24, 33]. However, Tween 20 used as a non-ionic surfactant exhibited permeation enhancer effect [34] having an impact on the improvement of the oral delivery of the drugs. Blank SEDDS formulations showed a mean droplet size below 150 nm and PDI less than 0.5 indicating the stable formulations as shown in Table 4. There is minor change in the size of the formulations after the incorporation of HIPs of TOB with DOC at the concentration of 1% w/v as shown in Table 5. A pay load of 1% was chosen as 0.5–2% payload led to shown encouraging results in in vivo studies [35]. Zeta potential of the unloaded formulations decreased gradually after the incorporation of the HIPs. Minor changes in the size and zeta potential of all the formulations were observed over time as shown in Tables 5 and 6 which might be due to the arrangement of the hydrophilic and lipophilic structures in the SEDDS formulations till a stable configuration of the formulations occurred [8].

**Table 5:** Assessment of mean droplet size, PDI and zeta potential of unloaded SEDDS as function of time using light scattering measurements. Indicated values are means of at least three experiments ± SD.

	*Blank SEDDS*			
*Time (h)*	*Size (nm)*	*Zeta Potential (mV)*	*Size (nm)*	*Zeta Potential (mV)*
	*PDI 0*		*PDI 4*	
***Formulation***				
FI	62.34 ± 2.39	2.47 ± 0.72	67.08 ± 1.98	2.15 ± 1.31
	0.24		0.29	
FII	103.82 ± 1.52	-4.49 ± 1.03	110.4 ± 2.57	-1.91 ± 0.72
	0.18		0.20	
FIII	146.23 ± 1.20	-7.98 ± 1.15	148.47 ± 1.73	-2.93 ± 0.26
	0.35		0.59	

**Table 6:** Assessment of mean droplet size, PDI and zeta potential of HIPs of TOB with DOC loaded SEDDS at a concentration of 1% w/v as function of time using light scattering measurements. Indicated values are means of at least three experiments ± SD.

	*Loaded SEDDS*			
*Time (h)*	*Size (nm)*	*Zeta Potential (mV)*	*Size (nm)*	*Zeta Potential (mV)*
	*PDI 0*		*PDI 4*	
***Formulation***				
FI	71.81 ± 1.27	-3.87 ± 1.93	73.44 ± 2.35	-4.06 ± 0.89
	0.12		0.16	
FII	106.11 ± 3.43	-7.17 ± 2.58	113.28 ± 1.78	-6.36 ± 2.69
	0.29		0.23	
FIII	147.38 ± 1.94	-11.45 ± 2.27	150.09 ± 2.26	-8.76 ± 1.19
	0.31		0.27	

### 3.6. Dissociation studies

**Fig 5:**
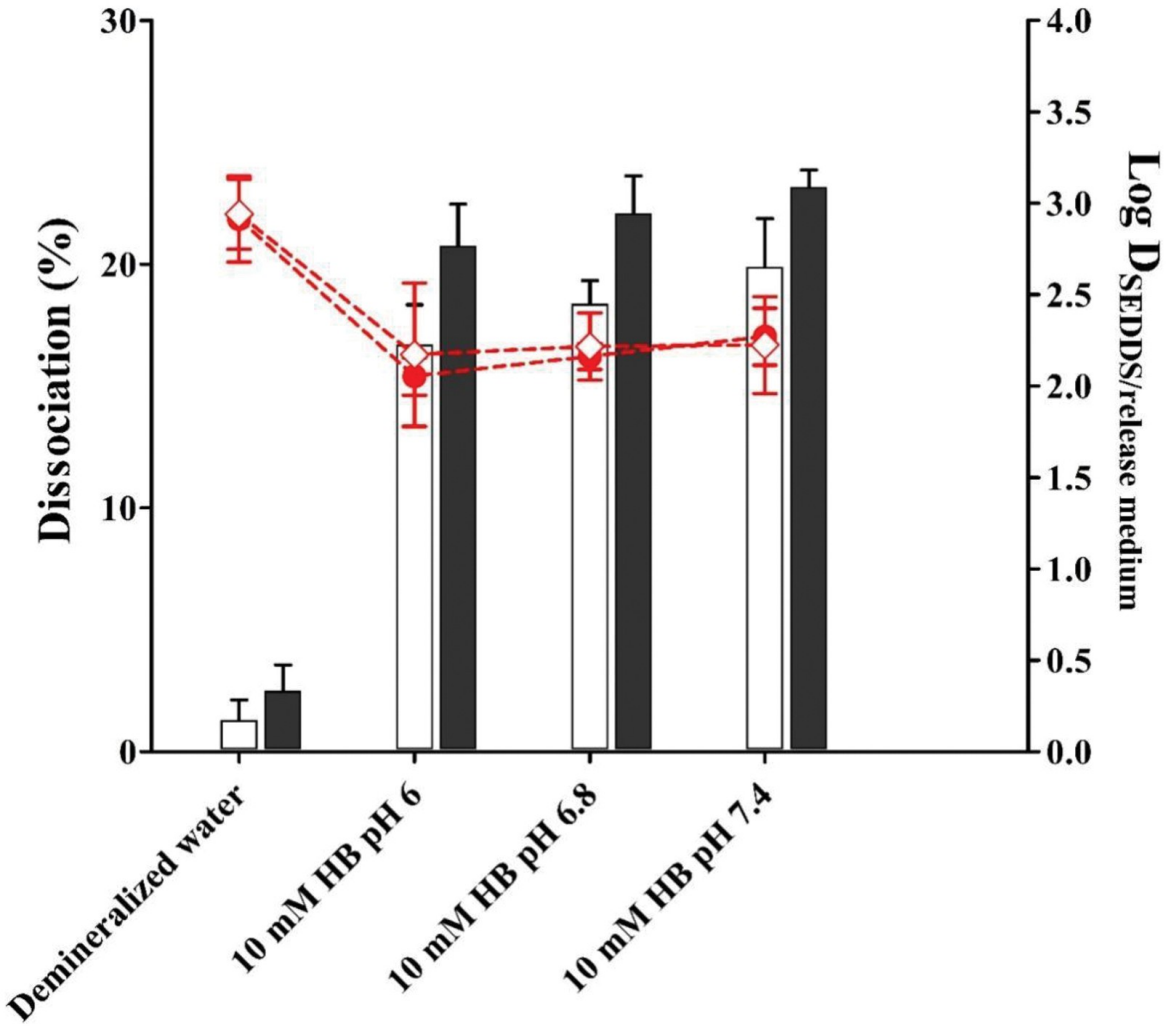
Dissociation of HIPs of TOB with DOC (1:5) at 2 h (white bars) and 4 h (black bars) at indicated pH. Log D_SEDDS/release medium_ of TOB-DOC at 2 h (red circle) and 4 h (red square). Indicated values are means of 3 experiments ± SD.

Results of dissociation of HIPs of TOB with DOC in demineralized water and in buffer at various pH conditions over time are illustrated in Fig 5. The higher stability of HIPs of TOB in water compared to the buffers could be related to the absence of ions in the medium, whereas factors as ionic strength and pH are not influencing the dissociation of HIPs. Dissociation of HIPs is improved when the complex is completely solubilized in co-solvents as a result of major interactions between water molecules and HIPs. The lipophilic complexes dissociated less than 30% at various intestinal pH conditions. These results indicated that HIPs of TOB remains in superassociated form during the permeation across the biological membranes.

### 3.7. Distribution coefficient (log D_SEDDS/release medium_) determination

Solubility of lipophilic complexes into SEDDS pre-concentrate was determined visually in order to define the maximum payload of drug to be incorporated into the formulation and calculate the distribution coefficient according to the dissociation study. The release of the incorporated lipophilic complex from the oily droplet is based on the simple diffusion process from the oily phase to the aqueous phase [17]. When the loaded HIPs release from the oily droplet and absorbed across the intestinal membrane, additional incorporated HIPs move out from the SEDDS until an equilibrium attained [36]. The solubility of HIPs in oily droplets was used for log D_SEDDS/release medium_ determination. Even if usually an optimum log D_SEDDS/release medium_ value should be comprised in the range from 3 to 5, when the purpose of HIPs incorporation into SEDDS is to increase the compound’s solubility, also log D_SEDDS/release medium_ values lower than 3 are acceptable because an immediate release of the compound from the systems seems to have a positive impact on drug’s solubility. As depicted in Fig 5, log D_SEDDS/release medium_ of HIPs of TOB was above 2 at various pH over time indicating that the lipophilic complexes are stable and retain in the oily droplets for extended time. The impact of SEDDS loaded with HIPs of BCS class III drug on the permeation across the intestinal membranes and bioavailability studies in vivo should certainly be investigated in future studies.

### 3.8. Cytotoxic potential determination-resazurin assay

**Fig 6:**
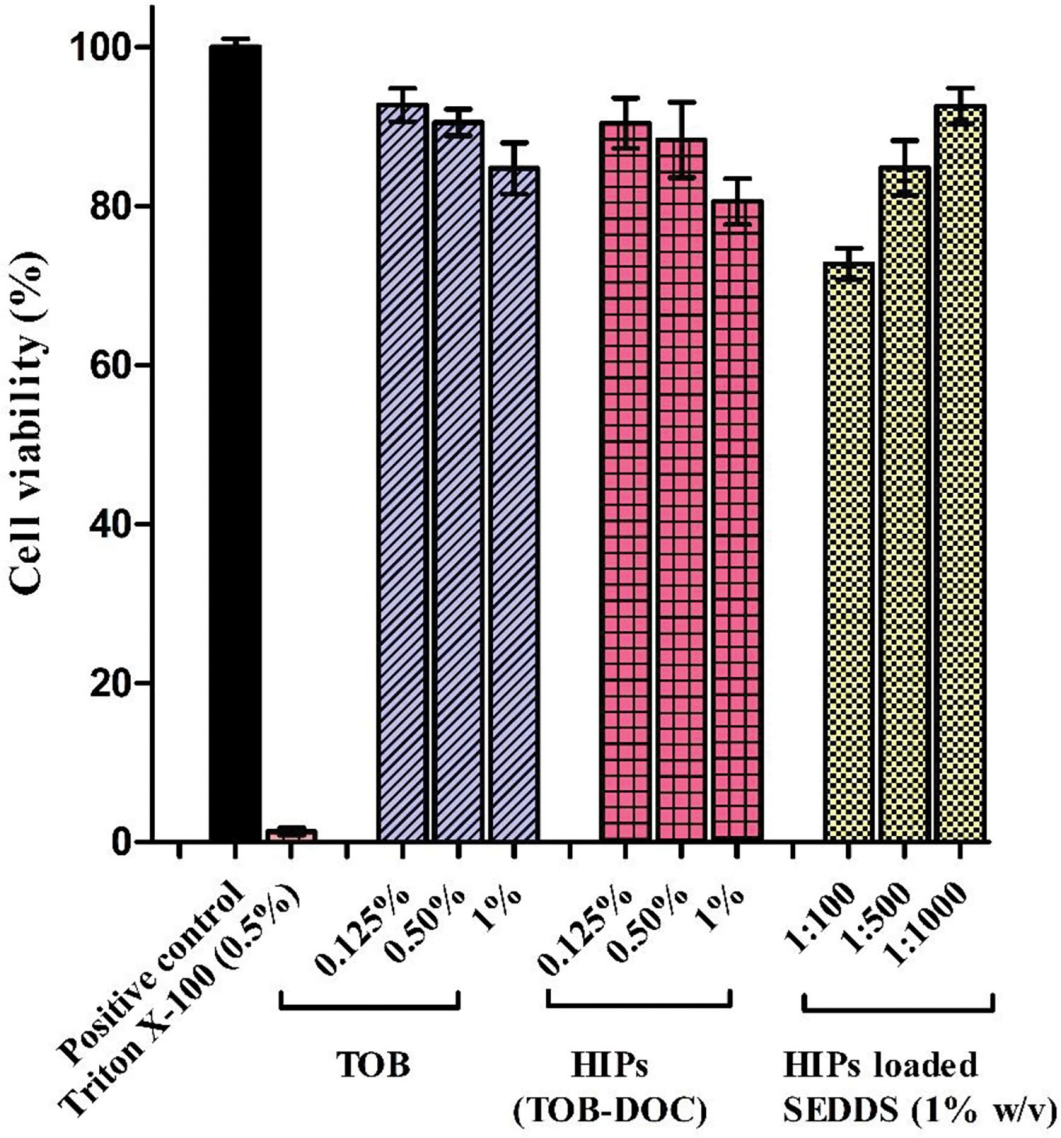
Impact of different concentration of TOB, HIPs of TOB and HIPs loaded SEDDS at various dilutions on the viability of Caco-2 cells after 4 h of incubation by means of resazurin assay. Indicated values are means of 3 experiments ± SD.

The resazurin assay was performed in order to evaluate the cytotoxic potential of TOB, HIPs of TOB with DOC and HIPs loaded SEDDS formulations. Cytotoxicity of the carrier systems considered as a basic parameter to envisage the destructive effects of these systems in vivo on the epithelial membranes [37]. The basic principle of resazurin assay is the capability of viable cells to convert resazurin into resorufin by reduction [38]. This compound, due to its fluorescence, can be quantified at an excitation wavelength (540 nm) and an emission wavelength (590 nm). Caco-2 cells were used because they can develop a monolayer of enterocytes, which are the main cells found in small intestine [39], [40]. Various concentration of TOB, TOB-DOC HIPs and TOB-DOC HIPs loaded SEDDS at a concentration of 1% w/v were tested. To prepare the samples the right percentage (w/v) of HIPs was dissolved in SEDDS and then loaded-SEDDS were solubilized in 10 mM HB at molar ratio 1:100, 1:500 and 1:1000. The composition of the buffer used in this experiment was the same of the one used for determining dissociation. As illustrated in **Fig 6**, various concentrations of TOB did not reduce the viability below the value of 80%. The reason of this behavior could be related to the fact that the formation of a neutral complex of TOB with the surfactant can reduce the presence of positive charge interacting with cell membranes and causing the toxicity.

## 4. Conclusions

Within the present study, we utilized the concept of HIP to improve the lipophilicity of a model BCS class III drug TOB followed by its incorporation into lipid based nanocarrier systems. Lipophilicity of TOB was increased utilizing various counterions. Lipid based delivery systems in particular SEDDS have synergistic effect as HIPs is likely stable in these oily droplets due to their lower dielectric constant compared to intestinal fluids and permeate more rapidly across the lipophilic membranes. Log D_SEDDS/release medium_ above 2 for all formulations demonstrated the stability of these lipophilic complexes in oily droplets in intestinal fluids as the release of HIPs from SEDDS is mainly controlled by log D_SEDDS/release medium_ value. Findings of this study suggested that HIP has shown to be a promising strategy for complexation of BCS class III drugs and their further incorporation into SEDDS may result in improve their oral bioavailability.
